# Branch Retinal Artery Occlusion as a Presentation of Seronegative Antiphospholipid Syndrome in Pregnancy

**DOI:** 10.7759/cureus.74198

**Published:** 2024-11-22

**Authors:** Joanne Shalini Chewa Raja, Muhammad Adib Redzuan, Abdullah Ashraf Rafique Ali

**Affiliations:** 1 Department of Ophthalmology, Faculty of Medicine, Universiti Teknologi MARA, Sungai Buloh, MYS; 2 Department of Ophthalmology, Hospital Al-Sultan Abdullah, Universiti Teknologi MARA, Puncak Alam, MYS

**Keywords:** antiphospholipid syndrome, branch retinal artery occlusion, ophthalmology, pregnancy, seronegative

## Abstract

A 40-year-old gravida 7 para 4+2 lady who was 14 weeks pregnant presented with a three-day history of sudden-onset flashes of light associated with a superonasal visual field defect on her right eye. She had two prior miscarriages that occurred in the second and third trimesters. Previous serological tests for antiphospholipid syndrome (APS) were normal. No other comorbidities were reported. On examination, visual acuity for both eyes was 6/6 with normal intra-ocular pressure. There was an embolus lodged at the inferotemporal peripheral retinal artery of the right eye with adjacent pale and oedematous distal retina. This corresponded with a superonasal scotoma on the Humphrey 30-2 visual field test. Other examinations were unremarkable. The patient was referred to obstetrics, rheumatology, and neuro-medical teams for co-management. Full blood count, coagulation profile, auto-immune panel, and antiphospholipid serological screening test were normal. No abnormalities were seen on her electrocardiogram and echocardiogram. A magnetic resonance angiogram of the brain revealed significant bilateral internal carotid stenosis at C2 and C6 levels. However carotid artery assessment showed normal flow below the jawline. The patient was diagnosed with seronegative APS with right-eye inferotemporal branch retinal artery occlusion (BRAO). A multidisciplinary team monitored her pregnancy, and she was started on subcutaneous enoxaparin 40 mg twice a day and oral aspirin/glycine 100 mg/45 mg once daily. At 20 weeks of pregnancy, the Hollenhorst plaque disappeared, and the visual field defect started to improve. She went on to deliver a healthy child at 36+6 weeks. Post-partum, she has continued on oral aspirin 100 mg once daily. A carotid angiogram performed post-delivery showed no evidence of internal carotid artery stenosis or thrombosis. This case highlights the importance of recognizing BRAO as it can be the presentation of a previously unrecognized seronegative antiphospholipid syndrome. Early recognition of the correct diagnosis is vital to allow effective multidisciplinary management, especially when dealing with a patient in pregnancy.

## Introduction

Branch retinal artery occlusion (BRAO) is a rare cause of visual loss that mainly occurs in the elderly [1]. Pregnancy is one of the risk factors for developing BRAO, where it might arise spontaneously from the physiologic adaptive changes, or it might be caused or aggravated by a co-existing underlying condition [2]. The presence of BRAO in a pregnant patient should prompt an investigation to identify the underlying cause. In this case report, we describe a pregnant lady who presented with right eye BRAO and was eventually diagnosed as having seronegative antiphospholipid syndrome (APS). To the best of our knowledge, this is the first report on BRAO during pregnancy that led to the diagnosis of seronegative APS.

## Case presentation

A 40-year-old gravida 7 para 4+2 lady who is pregnant at 14 weeks presented with a three-day history of sudden-onset flashes of light associated with a superonasal visual field defect on her right eye. She denied any floaters, and her central vision was not affected. She is a non-smoker. She claimed to have occasional migraine headaches that were not related to the current ocular symptoms. Previously, she had two prior miscarriages that occurred during the second and third trimesters, and anti-phospholipid antibody screening done at that time showed negative results. One week before her ocular symptoms, she was started on oral aspirin 150 mg once daily due to her previous history of miscarriages. No other comorbidities were reported.

**Figure 1:**
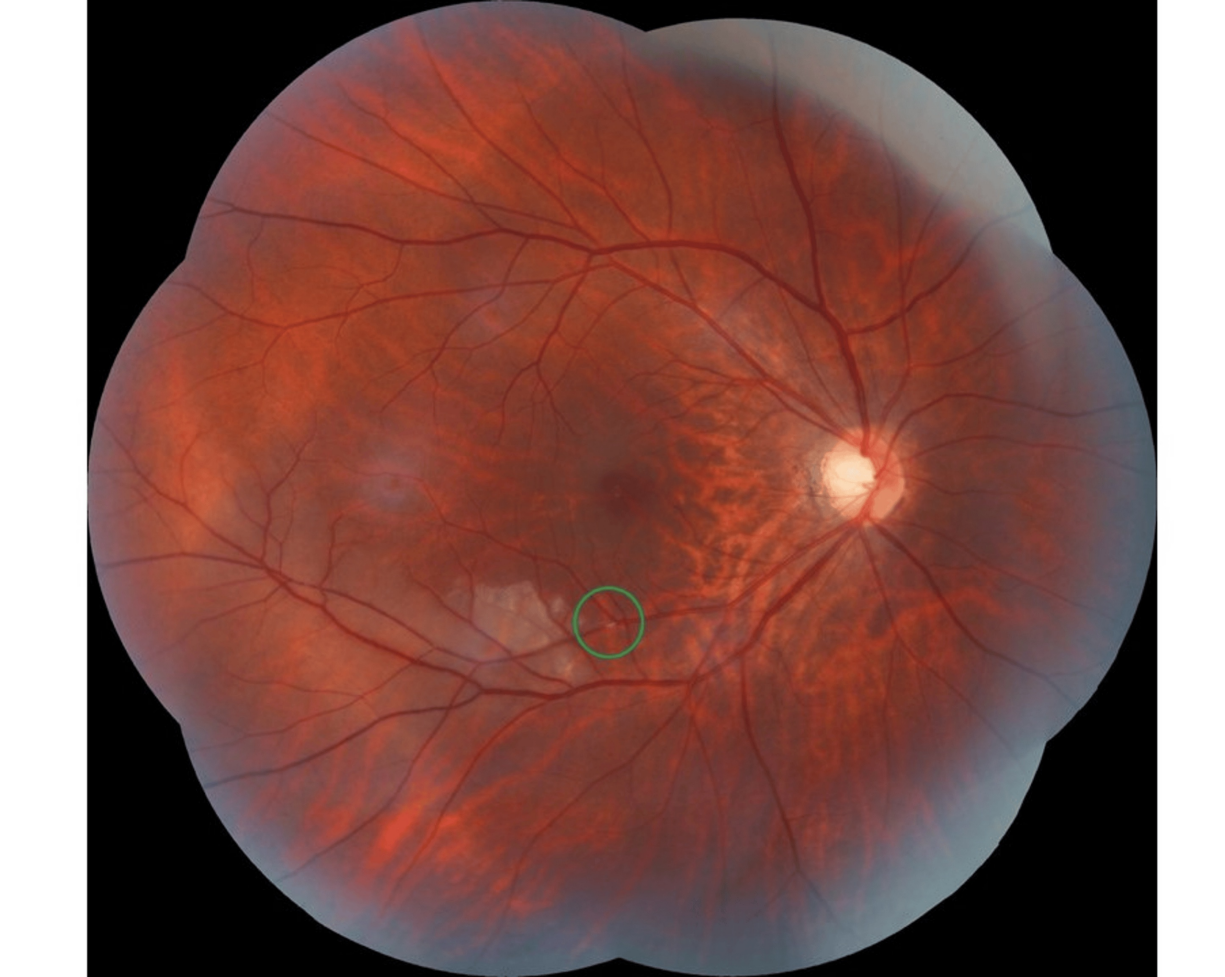
White cholesterol embolus (Hollenhorst plaque) lodged at the inferotemporal peripheral retinal artery of the right eye (green circle) with an adjacent pale and oedematous retina distal to the embolus.

**Figure 2:**
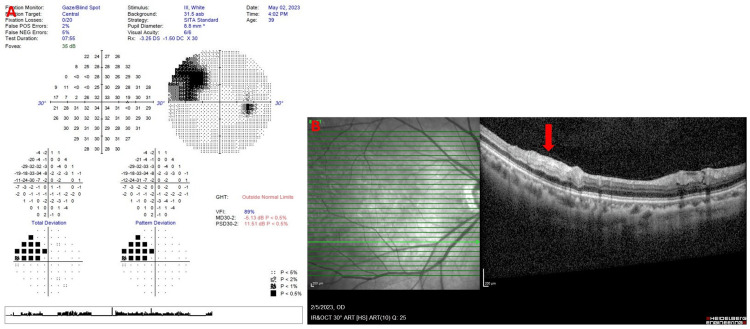
(A) A superonasal scotoma on the Humphrey 30-2 visual field test of the right eye. (B) An optical coherence test of the right eye macula showed hyper-reflectivity and increased thickness of the inner retinal layer just distal to the embolus (red arrow).

On examination, her blood pressure was 146/72 mmHg with a heart rate of 97 beats per minute. Her dextrose was 4.5 mmol/L, and she had a body mass index of 22 kg/m^2^. Ocular examination showed normal visual acuity (VA) of 6/6 with an intra-ocular pressure of 14 and 15 on the right and left eyes, respectively. There was a white cholesterol embolus (Hollenhorst plaque) lodged at the inferotemporal peripheral retinal artery of the right eye with an adjacent pale and oedematous retina distal to the embolus (Figure 1). This corresponded with a superonasal scotoma on the Humphrey 30-2 visual field test (Figure 2A). An optical coherence test of the right eye macula showed hyper-reflectivity and increased thickness of the inner retinal layer just distal to the embolus (Figure 2B). No carotid bruit was heard on auscultation. The rest of the ocular and systemic examinations were unremarkable.

A diagnosis of right-eye inferotemporal branch retinal artery occlusion was made based on the ocular findings. We attempted to dislodge the embolus by performing ocular massage and asking the patient to hyperventilate by breathing into a bag; however, both measures failed to dislodge the embolus. She was then started on topical bimatoprost 0.01% once a day at night to reduce the intra-ocular pressure, hoping that it would help in dislodging the embolus.

The patient was immediately referred to the obstetrics, neuro-medical, and rheumatology teams for co-management and was admitted to the gynecology ward for further investigation. Full blood count, coagulation profile, renal profile, and liver function tests were within normal range. Fasting plasma glucose, fasting serum lipids, and C-reactive protein were also normal. The erythrocyte sedimentation rate was slightly elevated at 24 mm/hour but within the expected range seen in pregnancy. Anti-cardiolipin IgG/IgM/IgA antibodies, lupus anticoagulant, and β2-glycoprotein antibody tests were negative. Other autoimmune serology tests, such as c-ANCA, p-ANCA, anti-nuclear antibody, anti-dsDNA antibody, and rheumatoid factor, were also negative. The electrocardiogram and echocardiogram were normal. Magnetic resonance angiogram of the brain revealed significant (70%) bilateral internal carotid artery stenosis at C2 and C6 levels; however, carotid Doppler ultrasound reported patent bilateral common carotid arteries (CCA) and bilateral carotid bulb. There was no obvious echogenic thrombus or narrowing seen, and normal color signal and waveform were demonstrated in both CCAs. She was started on subcutaneous enoxaparin 40 mg twice a day and oral aspirin/glycine 100 mg/45 mg once a day.

A multidisciplinary meeting was convened to discuss further management of this case, attended by the obstetrics, rheumatology, ophthalmology, neuro-medical, radiology, and anesthesia teams. The meeting discussed whether to proceed or terminate the pregnancy based on the patient’s history and presentation, due to the increased risk of stroke and other obstetrics complications, such as fetal growth restriction and hypertensive disorders of pregnancy. It also discussed the optimization of treatment and timing of delivery. During the meeting, it was agreed with the patient to proceed with the pregnancy, aiming for an early delivery via cesarean section at around 34-36 weeks, and to continue subcutaneous enoxaparin 40 mg twice a day and oral aspirin/glycine 100 mg/45 mg once daily.

**Figure 3:**
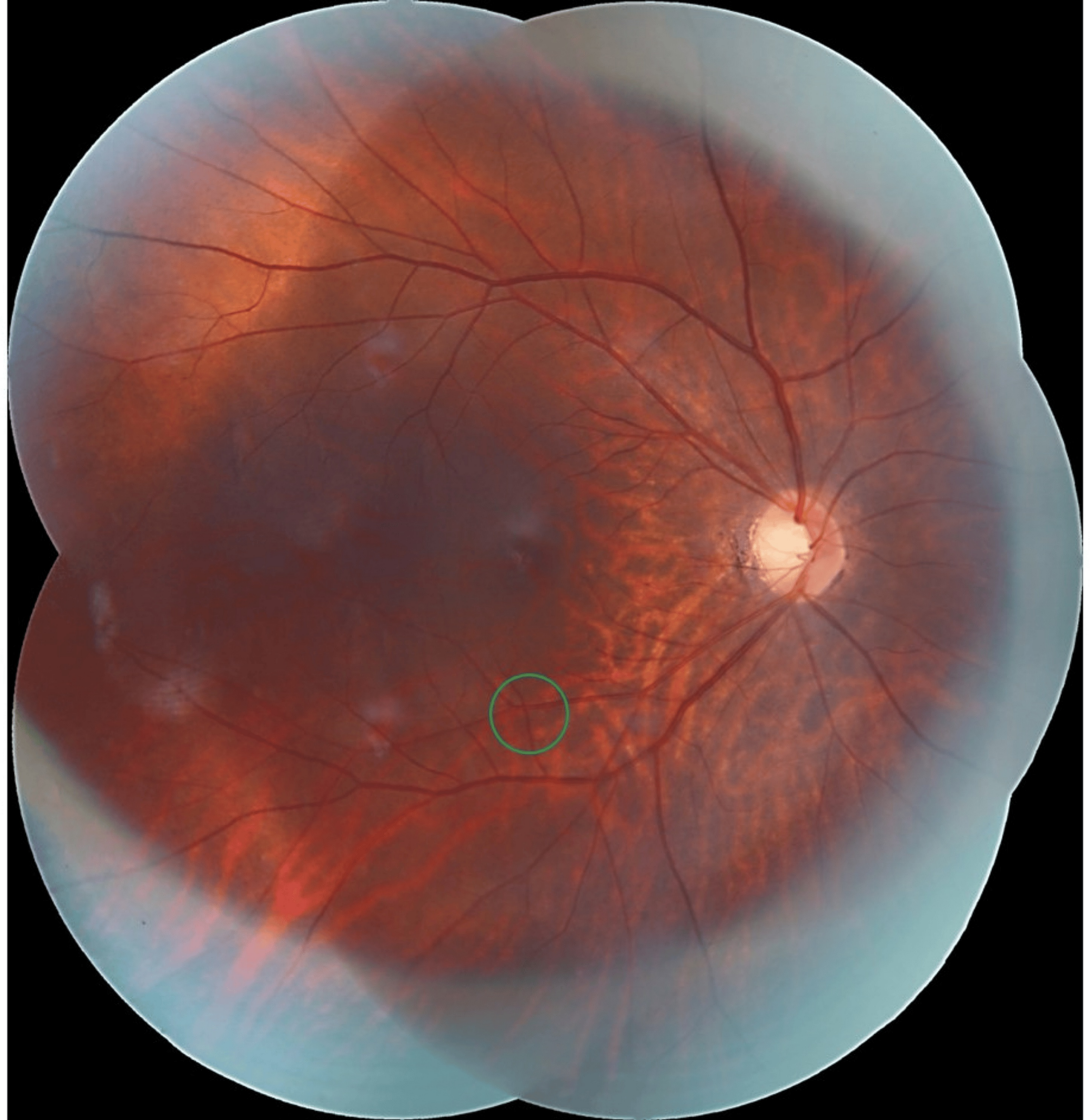
Fundus photography at three months post onset of right-eye inferotemporal BRAO - disappearance of the cholesterol emboli (green circle).

**Figure 4:**
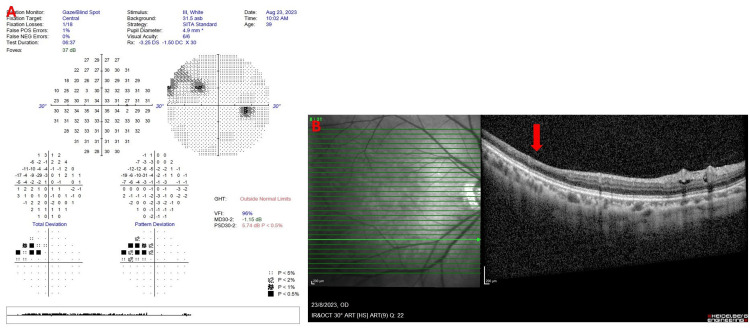
(A) Improvement of the superonasal scotoma on the right eye. (B) Thinning of the inner retinal layers distal to the site of the previous emboli (red arrow).

She was diagnosed with seronegative anti-phospholipid syndrome due to a history of recurrent miscarriage and right-eye branch retinal artery occlusion without any positive serological tests. Her pregnancy was closely monitored by a multidisciplinary team. At 20 weeks of pregnancy, the Hollenhorst plaque disappeared (Figure 3) with progressive improvement of the right-eye visual field defect (Figure 4A). The affected inner retinal layers started to become atrophied (Figure 4B). There was no evidence of any retinal neovascularization. At 36+6 weeks, she safely delivered a healthy baby girl weighing 2.82 kg with APGAR scores of 9 and 10 via elective cesarean section with bilateral fimbriectomy. Postpartum, she was continued on oral aspirin 100 mg once a day, and a carotid angiogram performed post-delivery showed no evidence of internal carotid artery stenosis or thrombosis.

## Discussion

Pregnancy induces several physiological changes to support the developing fetus and prepare the mother for labor and delivery. The cardiovascular system adapts to meet placental metabolic demands. The changes include increased cardiac output by 40% due to an increase in stroke volume and heart rate, while peripheral vasodilation leads to a 25-30% decrease in systemic vascular resistance. Alterations in the coagulation system during pregnancy produce a physiological hypercoagulable state (in preparation for hemostasis following delivery) [3]. The immunological system adapts to protect the mother and fetus while avoiding a fatal immunological response to the allogeneic fetus [3]. The above changes can increase the risk of retinal vascular occlusive events [4]. The incidence of retinal artery occlusion in pregnancy is also not well documented; in one retrospective case series of 147 patients who developed acute visual loss during pregnancy, 4.1% (n=2) were due to retinal artery occlusions [5].

APS is a systemic autoimmune condition characterized by recurrent vascular thrombosis involving both arteries and veins, fetal losses, and thrombocytopenia in the presence of antiphospholipid antibodies (aPL), including lupus anticoagulant (LA), anti-cardiolipin antibodies (aCL), and anti-β2 glycoprotein-I (anti-β2GPI) [6,7]. Seronegative APS was first defined by Shoenfeld et al. in 2003 as clinical manifestations highly suggestive of APS in the absence of laboratory criteria, such as LAC, aCL, and B2GP1 Abs [8]. Patients with a clinical history suggestive of APS, such as those with recurrent arterial venous thrombotic events, recurrent miscarriages, or unexplained thrombocytopenia, who have persistently negative aPL test results on at least two occasions, and who have had other causes of thrombosis ruled out, should be suspected of having seronegative APS, which is a diagnosis of exclusion [9]. In this case, apart from the BRAO, our patient had two prior miscarriages with twice negative aPL serology results, which were done during the current presentation and nine years prior.

APS can affect any part of the eye, including the anterior and posterior segments, as well as visual pathways in the central nervous system. However, involvement of the posterior segment of the eye is more common [10]. Reported BRAO cases associated with pregnancy in the literature are scarce. There are few cases where the diagnosis of BRAO has led to the diagnosis of a systemic condition. Kurtz et al. reported a case of unilateral BRAO during pregnancy that led to a previously undetected diagnosis of familial thrombophilia [11]. Askim et al. reported a case of a BRAO during pregnancy that subsequently led to the diagnosis of hereditary hemorrhagic telangiectasia [12]. This highlights the importance of proper history taking and workup in young pregnant patients presenting with BRAO, even though pregnancy itself is a risk factor like in the case we presented. Aggressive ocular management of BRAO is rarely pursued as prolonged ischemia often produces irreversible damage, and many occurrences of BRAO improve spontaneously [13].

Management of these cases requires a multidisciplinary approach as highlighted in this case, to avoid further thromboembolic events and ensure safe delivery of the baby and the mother's well-being. In general, the treatment of seronegative APS in pregnancy is similar to pregnant patients with seropositive APS. Low-dose aspirin (75-100 mg) is usually recommended in women with obstetrics APS without any thromboembolic or pregnancy complications. In the presence of any complications, the addition of low-molecular-weight heparin (LMWH) is recommended for these patients [14]. In this case, our patient completed her pregnancy without any further complications while being treated with both low-dose aspirin and LMWH.

## Conclusions

BRAO is one of the causes of visual loss and disturbances in pregnant women. This case highlights the importance of early recognition of BRAO as it can be the presentation of a previously unrecognized seronegative APS, especially in women with a history of previous miscarriages. Early recognition of the correct diagnosis is vital to allow effective multidisciplinary management, especially when dealing with a patient in pregnancy to ensure the safety of both the mother and the child.
